# Efficiency of Dinucleosides as the Backbone to Pre-Organize Multi-Porphyrins and Enhance Their Stability as Sandwich Type Complexes with DABCO

**DOI:** 10.3390/molecules22071112

**Published:** 2017-07-06

**Authors:** Sonja Merkaš, Souhaila Bouatra, Régis Rein, Ivo Piantanida, Mladen Zinic, Nathalie Solladié

**Affiliations:** 1 CNRS, LCC (Laboratoire de Chimie de Coordination), 205 Route de Narbonne, 31077 Toulouse France and Université de Toulouse, UPS, INPT, 31077 Toulouse, France; sonja.merkas@novartis.com (S.M.); bouatra@ualberta.ca (S.B.); regis.rein@lcc-toulouse.fr (R.R.); 2 Laboratory of Supramolecular and Nucleoside Chemistry, Rudjer Boskovic Institute, Bijenicka cesta 54, HR-10002 Zagreb, Croatia; ivo.piantanida@irb.hr (I.P.); mladen.zinic@irb.hr (M.Z.)

**Keywords:** porphyrins, nucleosides, supramolecular complexes

## Abstract

Flexible linkers such as uridine or 2′-deoxyuridine pre-organize bis-porphyrins in a face-to-face conformation, thus forming stable sandwich complexes with a bidentate base such as 1,4-diazabicyclo[2.2.2]octane (DABCO). Increased stability can be even greater when a dinucleotide linker is used. Such pre-organization increases the association constant by one to two orders of magnitude when compared to the association constant of DABCO with a reference porphyrin. Comparison with rigid tweezers shows a better efficiency of nucleosidic dimers. Thus, the choice of rigid spacers is not the only way to pre-organize bis-porphyrins, and well-chosen nucleosidic linkers offer an interesting option for the synthesis of such devices.

## 1. Introduction

Chemistry “beyond the molecule” is expressed in nature by a number of relatively weak noncovalent interactions [1,2]. The three-dimensional structures of most biopolymers are controlled with noncovalent interactions, either between different parts of the same strand (as in protein α-helices) or between two separate strands (as in the DNA duplex and protein β-sheet). Only in the past two decades have scientists begun to develop ways of mimicking the natural light-harvesting complexes by noncovalent assembly of porphyrin units with the aim of obtaining favored spacing and orientation between the chromophores [3,4,5,6,7,8,9,10,11,12,13,14,15,16,17,18,19,20,21]. The construction of multi-chromophoric assemblies has led to a resurgence of interest in coordination chemistry due to the formation of ordered arrays directed through molecular recognition events [22,23,24,25,26]. As a module in the construction of supramolecular assemblies, porphyrins and metalloporphyrins can be exploited in two different ways: porphyrins can behave as donor building blocks insofar as they comprise *meso*-substituents, such as pyridyl groups, which can act as ligands that can suitably coordinate to metal cations, while metalloporphyrins can act as acceptor building blocks as soon as the metal atom inside the porphyrin core has at least one axial site available for coordination. The interest in metalloporphyrins lies in the ease of exchange of various metals and ligands, which allow spatial control of porphyrin macrocycles. The coordination bond formed by metalloporphyrins has been explored in understanding how metal–ligand interactions are directing the formation of supramolecular porphyrin arrays. In this respect, a vast array of supramolecular systems has been prepared, such as cyclic oligomers [7,15,27,28], linear oligomers [29,30,31] and polymers [32,33,34], squares [35], tapes [36,37] and other geometries [8,38]. Important work on nucleotide–porphyrin or DNA–porphyrin conjugates for self-assembly and multi-porphyrin arrays has also been done in the last decade [39].

For the last few years, we have focused on cofacial bis-porphyrin tweezers for host–guest interactions and investigated the possibility of obtaining self-coordinated molecular systems with predictable spectral and redox characteristics [40]. We report here the synthesis of a di-nucleotide bearing pendant porphyrins dedicated to adopting a pre-organized conformation with face-to-face porphyrins, and capable of self-organizing in a stable sandwich type complex with a bidentate base such as 1,4-diazabicyclo[2.2.2]octane (DABCO). Using a similar strategy as the one used in antisense research, an artificial nucleotidic backbone was built from modified deoxy-uridine units linked with a more rigid linker than the phosphodiester moieties found in natural oligonucleotides. Antisense research uses modified oligonucleotides, less flexible than natural strands, to pre-organize the system toward the obtainment of stable double helices between synthesized and natural oligonucleotides. A modified oligonucleotidic backbone was used here to target a parallel conformation of the porphyrins appended to each deoxy-uridine moiety. To provide a rigid environment for the porphyrins, the uridine units were coupled in 3′–5′ stepwise fashion using an ether–ester type spacer of suitable length, and porphyrins were anchored to the uridine by means of robust carbon–carbon bonds. This is a first step toward the elaboration of longer oligonucleotides with pendant porphyrins and pre-defined conformation.

## 2. Results and Discussion

### 2.1. Advantage of Nucleosidic Linkers for the Pre-Organization of Dimers and Formation of Highly Stable Complexes with DABCO

**Figure 1:**
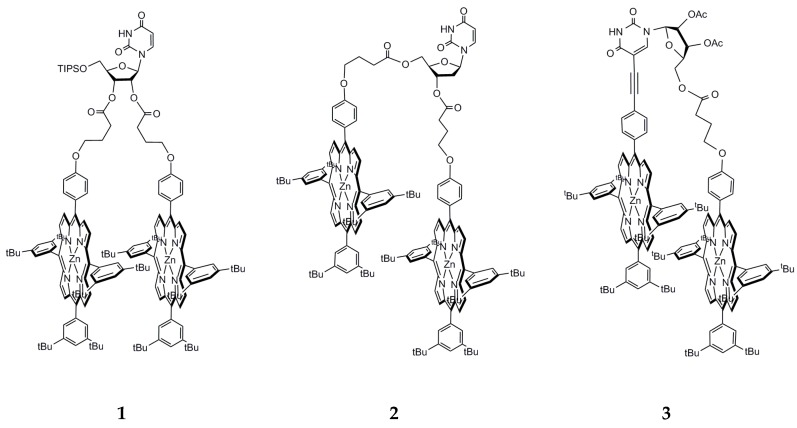
Three new bis-porphyrins bearing a flexible nucleosidic linker and differing in the attachment positions of the two porphyrins.

We reported a few years ago the binding studies of three bis-porphyrinic tweezers bearing nucleosidic linkers (Figure 1) [41]. These dimers differ in the attachment positions of the two porphyrins. For bis-porphyrinic dimers **1** and **2**, both porphyrins are attached to the ribose, in the O-2′ and O-3′ positions of uridine for dimer **1** and in the O-3′ and O-5′ positions of 2′-deoxyuridine for dimer **2**. For dimer **2** and **3**, the anchoring position of one Zn(II) porphyrin at the O-5′ position of the sugar moiety is conserved, while the second chromophore is attached either at the O-3′ position of the 2′-deoxyuridine for dimer **2** or at the C-5 position of the nucleic base for dimer **3** (Figure 1).

The ability of tweezers **1**, **2** and **3** to accommodate guests was investigated through binding studies carried out in dichloromethane with DABCO as a bidentate base. The coordination of DABCO was probed by UV-visible titration. It is based on the use of host–guest interactions for the formation of supramolecular assemblies between the multiporphyrinic arrays and small bidentate ligand (DABCO) through axial coordination. We chose DABCO as a ligand because its high basicity (pKa = 8.7) was expected to lead to stable complexes, although some other linear bidentate ligands would probably behave the same way. The complexations were monitored by UV-visible spectrophotometric titration of a solution of tweezers in CH_2_Cl_2_ [41]. The association constants for the formation of the complexes were calculated from UV-visible spectroscopic data. A value of 2.5 × 10^4^ M^−1^ (log K = 4.4) was found for tweezer **1**, which is similar to the association constant of DABCO with a reference Zn(II) porphyrin 5,10,15,20-tetra-di-*tert-*butyl-phenyl-porphyrin A_4_ (2.5 × 10^4^ M^−1^), thus indicating, as expected, that no well-defined, pre-organized conformation exists for bis-porphyrin **1**. However, much higher binding constants were found for the formation of host–guest complexes between tweezers **2** and **3** and DABCO. Values of 6.3 × 10^5^ M^−1^ (log K = 5.8) and 2.0 × 10^6^ M^−1^ (log K = 6.3) were found. These association constants are increased by 1.5 or 2 orders of magnitude as compared to the association constants of the same bidentate ligand with the above cited reference Zn(II) porphyrin. This enhanced stability of the complex may be ascribed to a pre-organization of the bis-porphyrinic tweezers **2** and **3** forming a cavity and provides convincing evidence that the bidentate base is inserted into the cavity of both dimers via host–guest interactions.

### 2.2. Comparison with the Pre-Organization Obtained When Rigid Linkers Are Used

**Figure 2:**
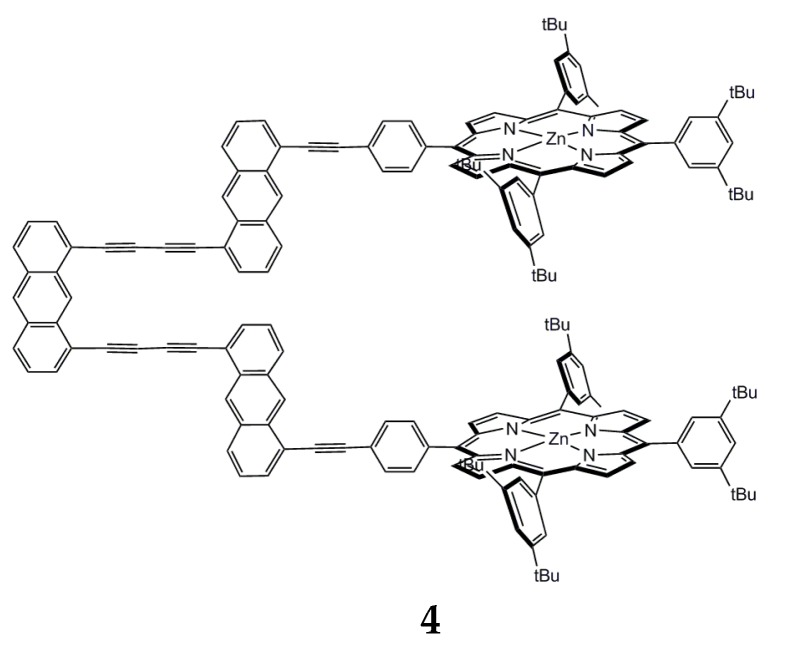
Rigid cofacial bis-porphrynic tweezers with tris-anthracenic spacer **4**.

To rationalize the pre-organization of these flexible dimers and their capability to behave as tweezers to complex bidentate guests, it is interesting to compare the obtained results with those of the rigid cofacial bis-porphyrinic tweezer **4** that we synthesized in a previous study (Figure 2) [12,41]. More specifically, this rigid dimer was designed in order to force the cofacial orientation of the porphyrins to create a cavity between the two chromophores, thus making them capable of hosting bidentate guests of appropriate size. A poly-anthracene was chosen as a rigid spacer for the construction of these tweezers. The tris-anthracenic spacer used in **4** forces a cofacial orientation of the chromophores while allowing a free rotation around the acetylenic axis, thus adjusting the cavity size to accommodate a large variety of guests. The ability of **4** to accommodate guests was investigated through binding studies carried out in dichloromethane with DABCO as a bidentate base. The complexations were monitored by UV-visible spectrophotometric titration in CH_2_Cl_2_. The association constant (K) between tweezer **4** and DABCO was calculated from the UV-visible spectroscopic data and a value of 3.9 × 10^5^ M^−1^ (log K = 5.6) was found. This association constant is increased by one order of magnitude, when compared to the association constant for the binding of the same bidentate ligands with a reference Zn(II) 5,10,15,20-tetra-di-*tert-*butyl-phenyl-porphyrin A_4_ (2.5 × 10^4^ M^−1^, log K = 4.4). The enhanced stability of the complex is ascribed to the pre-organization of the bis-porphyrinic tweezer **4**.

### 2.3. Dinucleotide: Formation of a Sandwich Type Molecular Complex with a Particularly High Association Constant

**Figure 3:**
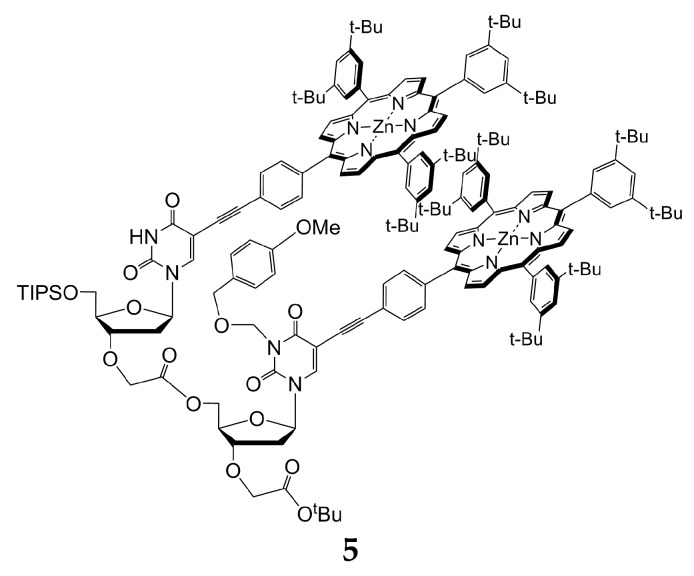
Flexible bis-porphrynic tweezers bearing a dinucleotidic spacer **5**.

The increased stability of the formation of the sandwich type host–guest complex with DABCO can be even greater when a dinucleotide linker is used (Figure 3). Such pre-organization increases the association constants by one to two orders of magnitude when compared to the association constants of the same bidentate ligands with a reference Zn(II) porphyrin. A comparison of these results with those obtained for the rigid tweezers **4** shows a better efficiency of the flexible nucleosidic dimers. We have observed and documented the fact that the choice of rigid spacers is not the only way to pre-organize bis-porphyrins, and that some well-chosen nucleosidic linkers offer an interesting option for the synthesis of such devices.

The ability of di-nucleotide **5** to accommodate guests was investigated through binding studies carried out in dichloromethane with DABCO as a bidentate base. As reported in some of our previous publications [42,43,44,45], it was assumed that the dinucleosidic bis-porphyrin could be pre-organized and thus facilitate the coordination of this rigid bifunctional ligand. The synthesized molecular system is amenable to preorganization, because its architecture is based on the combination of rigid and flexible linkers. The architecture of multi-porphyrinic systems is important because the covalent connectivity between the interacting centers commands the geometry of resulting assembly, and the relative orientation of chromophores dictates the strength of the interaction with ligands. As previously noted, the geometry of porphyrins in a supramolecular assembly is crucial in the design of artificial light-harvesting complexes [3,4,5,6,7,8,9,10,11,12,13,14,15,16,17,18,19,20,21]. Data obtained by UV-visible titrations were analyzed by using the values of the absorbance at fixed wavelengths, a method that is exclusively valid for the complexes with stoichiometry 1:1 [46], and by simultaneous fitting of the whole series of spectra collected at 1 nm intervals using the software SPECFIT (TgK Scientific, Bradford-on-Avon, UK) [47,48].

**Figure 4:**
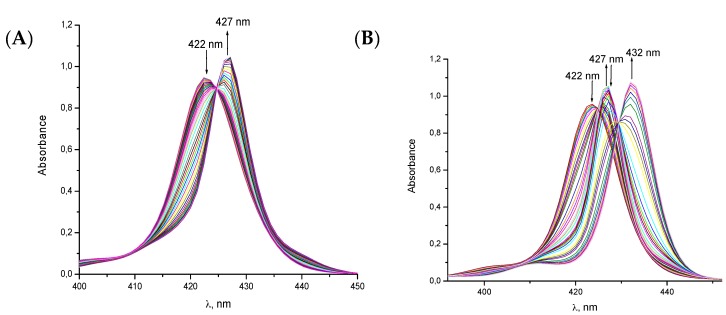
UV-visible titration of bis-porphyrinic tweezer **5** with 1,4-diazabicyclo[2.2.2]octane (DABCO) in CH_2_Cl_2_ at room temperature. (**A**) First part of the titration and (**B)** complete titration.

After examining the complexation of the simple porphyrin with DABCO, we studied the binding of the same base to dimer **5**, the bis-porphyrinic tweezer appended to an appropriate position of the flexible uridinic scaffold by the rigid acetylene group. The titration of the dimer with DABCO was measured by using the coordination shift of the Soret absorption as well as the Q-bands. The Soret band absorption was measured at the concentration of 1.0 × 10^−6^ M which corresponds to an absorbance of 0.95 at 422 nm. The titration was performed in two parts to be analyzed in terms of two-state equilibria. The first part of the titration was processed until two equivalents of DABCO were added per porphyrin. The complete titration that involved an addition of DABCO until 6000 equivalents per porphyrin was performed separately, as demonstrated in Figure 4.

**Figure 5:**
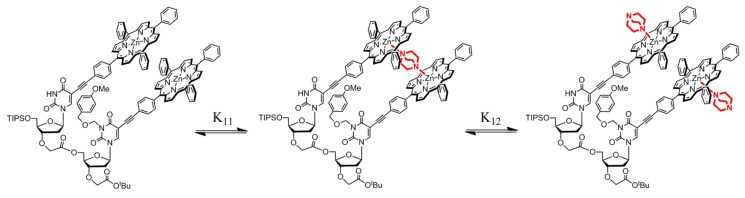
Representation of the proposed equilibria involved in the binding of DABCO to bis-porphyrinic tweezer **5**. For clarity, *tert*-butyls on *meso*-phenyls are omitted.

The clean isosbesticity of spectra obtained in the first part of the titration demonstrates that the only colored species present in the solution are free dimer **5** and one type of its complex with DABCO. The observed spectral changes are quite similar to those obtained in the titration of the monomer. However, there are some minor spectral changes, i.e., smaller red shift of the Soret peak, from 422 to 427 nm, as well as an intensified and sharpened form of the titration spectrum. A shift in the Soret band of 5 nm is exactly two times smaller than the shift obtained for the 1:1 monomer/DABCO complex. Accordingly, it was reasonable to anticipate formation of the intramolecular 1/1 sandwich complex. On the addition of more DABCO, the intensity of the absorption band at 427 nm decreased, and a new absorption band appeared at 432 nm. As observed in the titration spectrum of the monomer, the Soret absorption at 432 nm is typical for the simple 1:1 porphyrin/DABCO complex. On the basis of the changes in absorption spectra, the two possible consecutive two-state equilibria with corresponding stepwise constants in the binding of DABCO to bis-porphyrinic tweezer **5** are shown in Figure 5.

**Figure 6:**
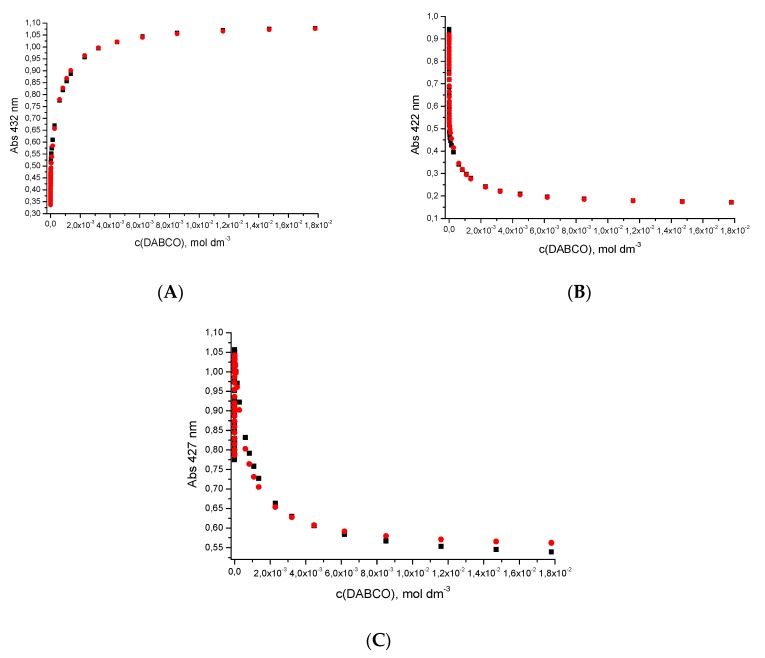
UV-visible titration data (change in absorbance at three wavelengths: (**A**) 432 nm, (**B**) 422 nm and (**C**) 427 nm) for the binding of bis-porphyrinic tweezer **5** with DABCO, fitted to the calculated curves for the equilibria in Figure 5.

The first method for calculation of the binding constant by using titration data at selected wavelengths was applied only to the first part of the titration characterized by a sharp isosbestic point. This analysis could be performed because the complexation by the 1:1 binding model was envisaged in the first equilibrium. The binding constant for the 1:1 dimer/DABCO complex was found to be 7.4 × 10^6^ M^−1^ (log K_11_ = 6.9). The formation of this type of a complex was verified by an analysis of the whole set of spectra which correspond to the first part of the titration by using SPECFIT. The analysis resulted in only one possible 1:1 complex with a corresponding binding constant log K_11_ = 6.42 ± 0.03. It is interesting to note that the calculated log K_11_ for the 1:1 dimer/DABCO complex is more than one order of magnitude higher than log K_11_ for the 1:1 monomer/DABCO complex. Taken together, these results strongly support the formation of a 1:1 dimer/DABCO sandwich complex through the chelate effect. The whole spectra obtained by the complete titration were analyzed in terms of three possible colored species: free dimer **5**, 1:1 sandwich complex, and 1:2 open complex (Figure 5). A fitting of the titration data revealed that only 1:1 and 1:2 binding models are possible for the dimer/DABCO complex, as shown in Figure 6.

Fitting to the 1:1 binding isotherm yielded the stoichiometric binding constant log K_11_ = 6.6, while the fit to the 1:2 binding model provided log K_12_ = 3.2. The calculated log K_11_ is almost identical to the log K_11_ value obtained in the analysis of the first part of the titration. Moreover, the calculated log K_12_ value is three orders of magnitude lower than the log K_11_ value. Therefore, dissociation of the more stable chelated 1:1 complex is followed by coordination of the second DABCO which is subsequently followed by the formation of the 1:2 complex in which two DABCO molecules are coordinated to two porphyrins of the dimer. Both 1:1 and 1:2 complexes involved in these consecutive two-state equilibria are in agreement with the species represented in Figure 5.

Similar formation of sandwich complexes was evidenced by Sanders, Hunter, Anderson and colleagues [49,50,51,52,53,54,55]. These studies show that sandwich complex formation is in concert with the parallel orientation of two porphyrin macrocycles. In this respect, the shifts in the titration spectra are caused by proximity of the porphyrins, and in this line, increased interporphyrinic electronic interaction. The Soret band of the free dimer **5** has a full width at half maximum (fwhm) of 15 nm, and the Soret band of the dimer/DABCO sandwich complex is 11 nm. It is believed that this narrowing indicates a significant increase in the center-to-center distance between the two porphyrins, due to the insertion of DABCO into the bis-porphyrinic cavity. This hypothesis is in great agreement with the full width at half maximum of 13 nm for the 1/2 dimer DABCO open complex. The outer coordination of DABCO allows a shorter center-to-center distance between the porphyrins.

## 3. Materials and Methods

Analyses by thin layer chromatography (TLC) were performed on a Merck silica-gel 60 F_254_ (Darmstadt, Germany), and TLC plaques were visualized by UV-lamp at wavelengths of 254 and 365 nm. Unless otherwise noted, for column chromatography Merck silica-gel 60 (40–63 μm or 63–200 μm) was used. For gel-permeation or size-exclusion column chromatography Bio-Beads S-X1 from Bio-Rad Laboratories (Richmond, CA, USA) were used in toluene.

The NMR spectra were recorded on Bruker spectrometers: Avance-300, ARX-250 and Avance 500 (Billerica, MA, USA) at the Laboratoire de Chimie de Coordination, Toulouse, France. UV-visible spectra were obtained using a Perkin Elmer Lambda 25 (Waltham, MA, USA) or on a Varian Cary 100 Bio (Palo Alto, CA, USA) spectrophotometer in dichloromethane. Fluorescence spectra were recorded on a Perkin Elmer LS 55 spectrometer in dichloromethane at the Laboratoire de Chimie de Coordination in Toulouse, France. Elemental analyses were carried out by the Service de microanalyse at the Laboratoire de Chimie de Coordination in Toulouse, France.

**Scheme 1:**
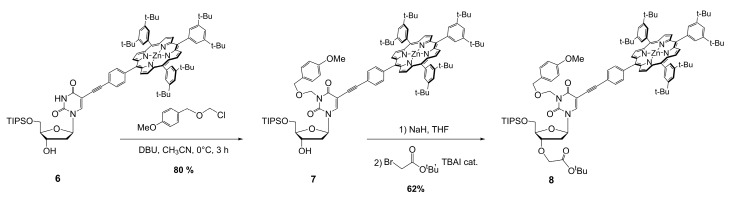
Synthesis of monomer **8**. DBU, 1,8-diazabicyclo[5.4.0]undec-7-ene; THF, tetrahydrofuran; TBAI, tetrabutylammonium iodide.

**Scheme 2:**
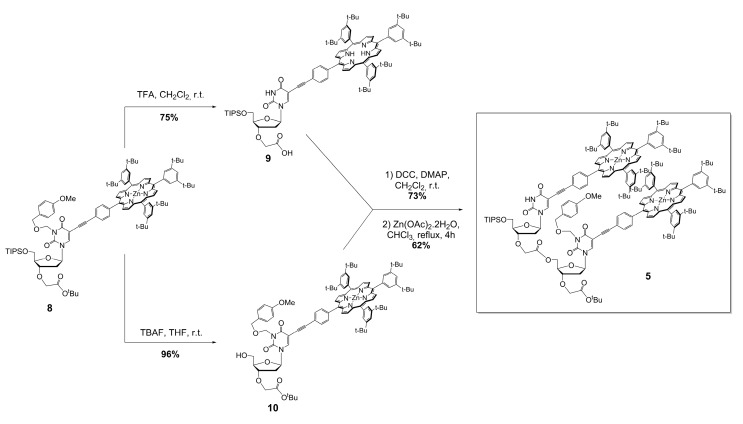
Synthesis of dimer **5**. TFA, trifluoroacetic acid; TBAF, tetrabutylammonium fluoride hydrate; DCC, *N*,*N*′-dicyclohexylcarbodiimide; DMAP, 4-(dimethylamino)pyridine.

The syntheses of monomer **8** and dimer **5** are summarized in Scheme 1 and Scheme 2 and described in the following sections.

### 3.1. Preparation of Porphyrin–Uridine Conjugate ***6***

The reaction was performed in four batches due to yields acquired at this scale. Thus, 5′-*O*-triisopropylsilyl-5-iodo-2′-deoxyuridine (0.31 g, 0.61 mmol) and 5-(4-ethynylphenyl)-10,15,20-tris(di-*tert*-butylphenyl)porphyrin metallated with Zinc(II) (0.77 g, 0.74 mmol) were dissolved in triethylamine (15 mL), freshly distilled and deoxygenized by bubbling with argon for 3 h. To this solution, Pd(PPh_3_)_2_Cl_2_ (0.011 g, 0.015 mmol) and CuI (0.006 g, 0.031 mmol) were added under an argon atmosphere. The mixture was stirred at room temperature (r.t.) for 60 h and then the batches were combined and concentrated under a vacuum. The dark brown residue was dissolved in dichloromethane and successively extracted with (ethylenedinitrilo)tetraacetic acid (EDTA) disodium salt (2% solution), Na_2_S_2_O_3_ (0.35 M), saturated solution of NH_4_Cl, and water. The organic phase was concentrated and SiO_2_ was added to the slurry, and then evaporated to dryness. The obtained composite was dried in a vacuum overnight and then purified by silica-gel column chromatography (Ø = 5 cm, h = 19 cm; hexane/ethyl acetate (8:2)), yielding compound **6** as a violet solid (3.07 g, 2.16 mmol, 88%). ^1^H-NMR (CDCl_3_, 500 MHz): δ = 9.00 (d, 2H, ^3^*J* = 4.5 Hz, H_β2_), 8.99 (s, 4H, H_β3_), 8.90 (d, 2H, ^3^*J* = 4.5 Hz, H_β1_), 8.21 (d, 2H, H_o_, ^3^*J* = 8.5 Hz), 8.13 (s, 1H, H_6_), 8.09 (dd, 6H, *J* = 2.0 and 4.0 Hz, H_o’’_), 7.87 (d, 2H, ^3^*J* = 8.0 Hz, H_m_), 7.79 (td, 3H, ^4^*J* = 2.0 and 6.5 Hz, H_p’+p’’_), 6.30 (dd, 1H, H_1’_, ^3^*J* = 6.5 and 7.0 Hz), 4.62 (m, 1H, H_3’_), 4.03 (m, 1H, H_4’_), 3.97 (m, 2H, H_5’_), 2.45 (ddd, 1H, H_2’_, ^3^*J* = 3.1 and 6.0 Hz and ^2^*J* = 13.6 Hz), 2.26 (ddd, 1H, H_2’_, ^3^*J* = 5.8 and 7.3 Hz and ^2^*J* = 13.9 Hz), 1.53 (s, 36H, *t*Bu), 1.52 (s, 18H, *t*Bu), 1.26 (s, 3H, CH_TIPS_), 1.15 (d, 9H, CH_3,TIPS_, ^3^*J* = 3.5 Hz), 1.13 (d, 9H, CH_3,TIPS_, ^3^*J* = 3.6 Hz). Elemental analysis (%) for C_88_H_106_N_6_O_5_SiZn (1421.30): calcd. C 74.36, H 7.52, N 5.91; found C 74.21, H 7.72, N 5.21. UV-visible (CH_2_Cl_2_): λabs (ε) = 423 nm (569,120 M^−1^ cm^−1^), 549 nm (21,793 M^−1^ cm^−1^), 589 nm (6173 M^−1^ cm^−1^).

### 3.2. Preparation of Porphyrin–Uridine Conjugate Protected at N-3 ***7***

Porphyrin–uridine conjugate **6** (1.00 g, 0.70 mmol) was suspended in acetonitrile (50 mL) and the obtained mixture was allowed to stir for 15 min. This homogeneous solution was deaerated and flushed with argon, and then cooled down to 0 °C. To this mixture, 1,8-diazabicyclo[5.4.0]undec-7-ene (DBU) (0.23 mL, 1.55 mmol) was added and then chloromethyl *p*-metoxybenzyl ether (0.26 mL, 1.41 mmol) was added in portions under an argon atmosphere. After an addition was completed, the solution was stirred for another 3 h at 0 °C. The product formation was followed by TLC in hexane/ethyl acetate (5:5) as an eluent, retention factor *R*_f_ = 0.52. After the reaction mixture was concentrated, the residue was dissolved in dichloromethane. This solution was washed with three portions of water, then evaporated and dried in a vacuum overnight. The crude product was purified by column chromatography (SiO_2_; hexane/ethyl acetate (from 8:2 to 2:8)), yielding the compound **7** as a violet solid (0.88 g, 0.56 mmol, 80%). ^1^H-NMR (CDCl_3_, 300 MHz): δ = 9.02 (d, 2H, ^3^*J* = 4.6 Hz, H_β2_), 9.00 (s, 4H, H_β3_), 8.92 (d, 2H, ^3^*J* = 4.7 Hz, H_β1_), 8.22 (d, 2H, H_o_, ^3^*J* = 8.2 Hz), 8.13 (s, 1H, H_6_), 8.09 (d, 4H, ^4^*J* = 1.8 Hz, H_o’’_), 8.08 (d, 2H, ^4^*J* = 1.8 Hz, H_o’_), 7.89 (d, 2H, ^3^*J* = 8.2 Hz, H_m_), 7.79 (td, 3H, ^4^*J* = 1.8 and 4.8 Hz, H_p’+p’’_), 7.38 (d, 2H, ^3^*J* = 8.8 Hz, H_d_), 6.90 (d, 2H, ^3^*J* = 8.8 Hz, H_c_), 6.30 (t, 1H, H_1’_, ^3^*J* = 6.5 Hz), 5.55 (s, 2H, H_a_), 4.71 (s, 2H, H_b_), 4.60 (m, 1H, H3’), 4.00 (ABM, 2H, H_5’_, ^3^*J* = 1.9 and 2.4 Hz and ^2^*J* = 8.9 Hz), 3.99 (m, 1H, H_4’_), 3.82 (s, 3H, H_e_), 2.46 (ddd, 1H, H_2’_, ^3^*J* = 3.0 and 5.7 Hz and ^2^*J* = 13.5 Hz), 2.20 (ddd, 1H, H_2’_, ^3^*J* = 6.0 and 7.7 Hz and ^2^*J* = 13.8 Hz), 1.54 (s, 36H, *t*Bu), 1.53 (s, 18H, *t*Bu), 1.26 (s, 3H, CH_TIPS_), 1.18 (d, 9H, CH_3_,_TIPS_, ^3^*J* = 3.5 Hz), 1.15 (d, 9H, CH_3_,_TIPS_, ^3^*J* = 3.5 Hz). Elemental analysis (%) for C_97_H_116_N_6_O_7_SiZn (1571.47): calcd. C 74.14, H 7.44, N 5.35; found C 73.41, H 7.56, N 5.08 (+1 ethyl acetate).

### 3.3. Preparation of Monomer ***8***

The whole system—a round-bottom flask equipped with a magnetic stirring bar and an addition funnel—was extra dried, deaerated and flushed with argon. The suspension of NaH (0.005 g, 0.12 mmol) in dry tetrahydrofuran (THF, 2.0 mL) was cooled using an ice–salt bath, and then charged with the uridine–porphyrin conjugate **7** (0.10 g, 0.064 mmol). The mixture was stirred at 0 °C for 1 h and then at r.t. for 4 h to complete deprotonation. Again, the reaction was cooled using an ice–salt bath and charged with tetrabutylammonium iodide (TBAI) (0.007 g, 0.0191 mmol), and then an additional funnel was filled with the solution of *tert*-butyl 2-bromoacetate (0.012 mL, 0.083 mmol) in dry THF (0.1 mL), which was added dropwise into the reaction under an argon atmosphere. The mixture was then stirred overnight, allowing it to slowly warm to r.t. The product formation was followed by TLC in hexane/ethyl acetate (7.5:2.5) as an eluent, *R*_f_ = 0.40. Removal of solvent via rotary evaporation afforded residue which was then dissolved in dichloromethane. The corresponding solution was washed with one portion of saturated solution of NH_4_Cl and two portions of water, before it was evaporated to dryness. The crude product was purified by using column chromatography (SiO_2_; hexane/ethyl acetate (from 9:1 to 6:4)), to yield monomer **8** as a violet solid (0.067 g, 0.040 mmol, 62%) together with residual unreacted 6 (0.03 g, 0.020 mmol, 29%). ^1^H-NMR (CDCl_3_, 300 MHz): δ = 9.02 (d, 2H, ^3^*J* = 4.4 Hz, H_β2_), 9.01 (s, 4H, H_β3_), 8.92 (d, 2H, ^3^*J* = 4.7 Hz, H_β1_), 8.21 (d, 2H, H_o_, ^3^*J* = 8.4 Hz), 8.19 (s, 1H, H_6_), 8.09 (d, 4H, ^4^*J* = 2.0 Hz, H_o”_), 8.08 (d, 2H, ^4^*J* = 2.0 Hz, H_o’_), 7.88 (d, 2H, ^3^*J* = 8.2 Hz, H_m_), 7.79 (td, 3H, ^4^*J* = 1.8 and 4.7 Hz, H_p’+p’’_), 7.39 (d, 2H, ^3^*J* = 8.8 Hz, H_d_), 6.91 (d, 2H, ^3^*J* = 8.7 Hz, H_c_), 6.38 (dd, 1H, H_1’_, ^3^*J* = 7.9 and 5.7 Hz), 5.57 (s, 2H, H_a_), 4.72 (s, 2H, H_b_), 4.34 (m, 1H, H_3’_), 4.30 (m, 1H, H_4’_), 4.06 (dd, 2H, H_5’_, ^3^*J* = 2.0 and 3.0 Hz and ^2^*J* = 9.0 Hz), 4.04 (AB, 2H, CH_2_, ether, ^2^*J* = 16.38 Hz), 3.82 (s, 3H, H_e_), 2.63 (ddd, 1H, H_2’_, ^3^*J* = 1.9 and 5.6 Hz and ^2^*J* = 13.8 Hz), 2.12 (ddd, 1H, H_2’_, ^3^*J* = 6.6 and 8.1 Hz and ^2^*J* = 14.4 Hz), 1.53 (s, 36H, *t*Bu), 1.52 (s, 18H, *t*Bu), 1.51 (s, 9H, *t*Bu), 1.26 (s, 3H, CH_TIPS_), 1.18 (d, 9H, CH_3,TIPS_, ^3^*J* = 3.3 Hz), 1.16 (d, 9H, CH_3,TIPS_, ^3^*J* = 3.4 Hz). Elemental analysis (%) for C_103_H_126_N_6_O_9_SiZn (1685.61): calcd. C 73.39, H 7.53, N 4.99; found C 73.13, H 7.44, N 4.62.

### 3.4. Preparation of Monomer-Acid ***9***

Monomer **8** (1.00 g, 0.59 mmol) was dissolved in CH_2_Cl_2_ and stabilized with amylene (25 mL) and the solution was cooled using an ice–salt bath, and then TFA (5.0 mL, 0.065 mol) was added. The mixture was flushed with argon, and then left to stir. After 1 h at 0 °C, the bath was removed and the mixture was allowed to warm to r.t. The reaction was stirred for 5 h at r.t.; then it was analysed completely by TLC analysis in the mixture of ethyl acetate/methanol (8.5:1.5) with a few drops of acetic acid (*R*_f_ (**9**) = 0.48). The reaction solution was diluted and successively washed with water until the pH of the aqueous phase was adjusted to 8. The solvent from the organic phase was removed by evaporation, and subsequently, the crude product was dried in a vacuum overnight. Finally, the pure product was isolated by silica-gel column chromatography (ethyl acetate +0.1% of acetic acid and gradually increased methanol up to 5%), to yield monomer-acid **9** as a violet solid (0.63 g, 0.445 mmol, 75%). ^1^H-NMR (CDCl_3_, 300 MHz): δ = 8.90 (d, 2H, ^3^*J* = 4.3 Hz, H_β2_), 8.89 (s, 4H, H_β3_), 8.80 (d, 2H, ^3^*J* = 4.8 Hz, H_β1_), 8.21 (d, 2H, H_o_, ^3^*J* = 8.0 Hz), 8.22 (s, 1H, H_6_), 8.08 (d, 4H, ^4^*J* = 1.8 Hz, H_o’’_), 8.07 (d, 2H, ^4^*J* = 2.2 Hz, H_o’_), 7.88 (d, 2H, ^3^*J* = 8.2 Hz, H_m_), 7.79 (td, 3H, ^4^*J* = 1.7 and 3.3 Hz, H_p’+p’’_), 6.38 (dd, 1H, H_1’_, ^3^*J* = 5.7 and 8.0 Hz), 4.39 (m, 1H, H_3’_), 4.31 (m, 1H, H_4’_), 4.23 (s, 2H, CH_2_, ether), 4.06 (ABM, 2H, H_5’_, ^3^*J* = 1.4 and 3.0 Hz and ^2^*J* = 9.6 Hz), 2.67 (ddd, 1H, H_2’_, ^3^*J* = 1.5 and 5.6 Hz and ^2^*J* = 13.3 Hz), 2.18 (ddd, 1H, H_2’_, ^3^*J* = 6.4 and 7.9 Hz and ^2^*J* = 14.3 Hz), 1.52 (s, 54H, *t*Bu), 1.26 (s, 3H, CH_TIPS_), 1.19 (d, 9H, CH_3,TIPS_, ^3^*J* = 3.6 Hz), 1.16 (d, 9H, CH_3,TIPS_, ^3^*J* = 3.7 Hz), −2.7 (s, 2H, NH).

### 3.5. Preparation of Monomer-Alcohol ***10***

Monomer **8** (1.00 g, 0.59 mmol) was dissolved in THF (30 mL), and TBAF (2.0 mL, 1.19 mmol, 1 M solution in THF) was added. The mixture was deareated and flushed with argon, and then left to stir at r.t. for 5 h under an argon atmosphere. The formation of the product was followed by TLC in hexane/ethyl acetate (6.5:3.5) as an eluent, *R*_f_ = 0.44. The solvent was removed by evaporation and the residue was dissolved in dichloromethane. The obtained solution was washed with three portions of water, and the organic phase was removed under reduced pressure. Finally, the pure product was isolated by silica-gel column chromatography (hexane/ethyl acetate (8:2)), to afford monomer-alcohol **10** as a violet solid (0.86 g, 0.565 mmol, 96%). ^1^H-NMR (CDCl_3_, 300 MHz): δ = 9.05 (d, 2H, ^3^*J* = 4.1 Hz, H_β2_), 9.04 (s, 4H, H_β3_), 9.00 (d, 2H, ^3^*J* = 4.7 Hz, H_β1_), 8.25 (d, 2H, H_o_, ^3^*J* = 8.1 Hz), 8.13 (d, 4H, ^4^*J* = 1.8 Hz, H_o’’_), 8.12 (d, 2H, ^4^*J* = 1.8 Hz, H_o’_), 8.04 (s, 1H, H_6_), 7.94 (d, 2H, ^3^*J* = 8.1 Hz, H_m_), 7.83 (m, 3H, H_p’+p’’_), 7.36 (d, 2H, ^3^*J* = 6.9 Hz, H_d_), 6.89 (d, 2H, ^3^*J* = 7.0 Hz, H_c_), 6.04 (t, 1H, H_1’_, ^3^*J* = 6.4 Hz), 5.53 (s, 2H, H_a_), 4.70 (s, 2H, H_b_), 4.18 (td, 1H, H_3’_, ^3^*J* = 4.2 and 8.1 Hz), 4.00 (AB, 2H, CH_2_, ether, ^2^*J* = 16.5 Hz), 3.95–3.71 (m, 3H, H_4’_ + H_5’_), 3.81 (s, 3H, H_e_), 2.44 (ddd, 1H, H_2’_, ^3^*J* = 4.2 and 6.0 Hz and ^2^*J* = 13.5 Hz), 2.27 (td, 1H, H_2’_, ^3^*J* = 6.3 Hz and ^2^*J* = 13.2 Hz), 1.56 (s, 36H, *t*Bu), 1.55 (s, 18H, *t*Bu), 1.50 (s, 9H, *t*Bu).

### 3.6. Preparation of Free-Base Dimer

Monomer-alcohol **10** (0.66 g, 0.430 mmol) and monomer-acid **9** (0.61 g, 0.430 mmol) were dissolved in dichloromethane (without ethanol and dried over molecular sieves, 40 mL) and to this solution 4-(dimethylamino)pyridine (DMAP) (0.11 g, 0.903 mmol) and *N*,*N*′-dicyclohexylcarbodiimide (DCC, 0.12 g, 0.602 mmol) were added, respectively. The obtained mixture was allowed to stir at r.t. overnight under an argon atmosphere. The reaction progress was followed by a TLC analysis examining the disappearance of both the starting materials and formation of the product (hexane/ethyl acetate (6.5:3.5), *R*_f_ = 0.31). The solvent was removed to dryness via rotary evaporation and afforded residue was suspended in minimum volume of toluene. The slurry was placed in refrigerator for 3 h to precipitate dichlorolurea (DCU). The precipitated white solid was collected by filtration and washed with small portions of chilled toluene. The obtained filtrate was evaporated in vacuo and then left to dry in a vacuum overnight. The crude product was purified by using column chromatography (SiO_2_; hexane/ethyl acetate (from 8:2 to 6:4)) and preparative size exclusion chromatography (Bio-Beads, Bio-Rad Laboratoriestoluene), yielding the free-base dimer as a violet solid (0.92 g, 0.314 mmol, 73%). ^1^H-NMR (CDCl_3_, 500 MHz, c = 3.027 × 10^−3^ M): δ = 9.01 (d, 2H, ^3^*J* = 4.6 Hz, Zn-H_β2_), 8.99 (s, 4H, Zn-H_β3_), 8.92 (d, 2H, ^3^*J* = 4.7 Hz, Zn-H_β1_), 8.89 (s, 4H, 2H-H_β3_), 8.88 (d, 2H, ^3^*J* = 4.6 Hz, 2H-H_β2_), 8.76 (d, 2H, ^3^*J* = 4.7 Hz, 2H-H_β1_), 8.25 (d, 2H, Zn-H_o_, ^3^*J* = 8.5 Hz), 8.16 (d, 2H, 2H-H_o_, ^3^*J* = 8.0 Hz), 8.10 (t, 4H, ^4^*J* = 1.3 Hz, Zn-H_o’_), 8.08 (d, 2H, ^4^*J* = 1.8 Hz, Zn-H_o’_), 8.07 (d, 6H, ^4^*J* = 1.8 Hz, 2H-H_o’_), 8.06 (s, 1H, 2H-H_6_), 7.93 (d, 2H, ^3^*J* = 8.0 Hz, Zn-H_m_), 7.92 (s, 1H, Zn-H_6_), 7.78 (td, 5H, ^4^*J* = 1.5 and 3.5 Hz, H_p’_), 7.76 (t, 1H, ^4^*J* = 2.0 Hz, H_p’_), 7.75 (d, 2H, ^3^*J* = 8.0 Hz, 2H-H_m_), 7.39 (d, 2H, ^3^*J* = 9.0 Hz, H_d_), 6.92 (d, 2H, ^3^*J* = 8.5 Hz, H_c_), 6.31 (t, 1H, Zn-H_1’_, ^3^*J* = 6.5 Hz), 6.28 (dd, 1H, 2H-H_1’_, ^3^*J* = 8.0, ^4^*J* = 5.0 Hz), 5.57 (s, 2H, H_a_), 4.73 (s, 2H, H_b_), 4.55 (ABM, 2H, 2H-CH_2_, ether, ^3^*J* = 3.5 and 4.3 Hz and ^2^*J* = 7.8 Hz), 4.45 (q, 1H, Zn-H_4’_, ^3^*J* = 4.0 Hz), 4.41 (d, 1H, 2H-H_3’_, ^3^*J* = 5.5 Hz), 4.39 (s, 2H, Zn-CH_2_, ether), 4.31 (m, 1H, 2H-H_4’_), 4.29 (m, 1H, Zn-H_3’_), 4.07 (AB, 2H, Zn-H_5’_, ^2^*J* = 16.5 Hz), 4.02 (ABM, 2H, 2H-H_5’_, ^3^*J* = 1.0 and 1.8 Hz and ^2^*J* = 10.0 Hz), 3.82 (s, 3H, H_e_), 2.68 (ddd, 1H, 2H-H_2’_, ^3^*J* = 3.0 and 5.5 Hz and ^2^*J* = 14.3 Hz), 2.60 (ddd, 1H, Zn-H_2’_, ^3^*J* = 5.3 and 6.8 Hz and ^2^*J* = 13.8 Hz), 2.27 (dd, 1H, Zn-H_2’_, ^3^*J* = 7.5 Hz and ^2^*J* = 14.3 Hz), 2.11 (ddd, 1H, 2H-H_2’_, ^3^*J* = 6.2 and 8.6 Hz and ^2^*J* = 13.9 Hz), 1.53 (s, 72H, *t*Bu), 1.52 (s, 36H, *t*Bu), 1.51 (s, 9H, *t*Bu), 1.25 (s, 3H, CH_TIPS_), 1.12 (d, 9H, CH_3,TIPS_, ^3^*J* = 4.0 Hz), 1.11 (d, 9H, CH_3,TIPS_, ^3^*J* = 3.5 Hz), −2.70 (s, 2H, NH).

### 3.7. Preparation of Zinc(II) Porphyrin Dimer ***5***

The solution of Zn(OAc)_2_·2H_2_O (0.11 M) was prepared separately by dissolving the corresponding salt (0.0042 g, 0.019 mmol) in methanol (0.18 mL). Such solution was added to the mixture of a free-base dimer (0.028 g, 0.0096 mmol) in chloroform (7 mL), and the obtained homogenous mixture was flushed with argon and heated to reflux. The progress of metallation was monitored by UV-visible spectroscopy showing that the reaction was terminated after 4 h of reflux under an argon atmosphere. The solvent was removed by evaporation, the residue was dissolved in dichloromethane and the solution was washed with water. The crude product was purified by column chromatography (SiO_2_; hexane/ethyl acetate (7:3)) yielding **5** (0.018 g, 0.0059 mmol, 62%). ^1^H-NMR (CDCl_3_, 250 MHz): δ = 9.02 (d, 2H, ^3^*J* = 4.7 Hz, H_β2_), 9.00 (s, 8H, H_β3_), 8.99 (d, 2H, ^3^*J* = 4.7 Hz, H_β2_), 8.94 (d, 2H, ^3^*J* = 4.7 Hz, H_β1_), 8.87 (d, 2H, ^3^*J* = 4.7 Hz, H_β1_), 8.26 (d, 2H, H_o_, ^3^*J* = 8.2 Hz), 8.17 (d, 2H, H_o_, ^3^*J* = 8.1 Hz), 8.11 (d, 4H, ^4^*J* = 1.7 Hz, H_o’_), 8.09 (d, 8H, ^4^*J* = 1.7 Hz, H_o’_), 8.05 (s, 1H, H_6_), 7.94 (d, 2H, ^3^*J* = 8.2 Hz, H_m_), 7.91 (s, 1H, H_6_), 7.79 (s, 6H, H_p’_), 7.75 (d, 2H, ^3^*J* = 8.4 Hz, H_m_), 7.39 (d, 2H, ^3^*J* = 8.7 Hz, H_d_), 6.91 (d, 2H, ^3^*J* = 8.7 Hz, H_c_), 6.29 (t, 1H, H_1’_, ^3^*J* = 6.7 Hz), 6.26 (t, 1H, H_1’_, ^3^*J* = 6.9 Hz), 5.57 (s, 2H, H_a_), 4.73 (s, 2H, H_b_), 4.55 (ABM, 2H, CH_2_, ether, ^3^*J* = 3.8 and 5.1 Hz and ^2^*J* = 8.1 Hz), 4.42 (m, 2H, H_4’_+H_3_), 4.38 (s, 2H, CH_2_, ether), 4.30 (m, 2H, H_4’_+H_3_), 4.06 (AB, 2H, H_5’_, ^2^*J* = 14.2 Hz), 4.05 (m, 2H, H_5’_), 3.82 (s, 3H, H_e_), 2.64 (m, 2H, H_2’_), 2.27 (q, 1H, H_2’_, ^3^*J* = 7.0 Hz), 2.11 (m, 1H, H_2’_), 1.52 (s, 108H, *t*Bu), 1.50 (s, 9H, *t*Bu), 1.26 (s, 3H, CH_TIPS_), 1.14 (d, 9H, CH_3,TIPS_, ^3^*J* = 1.3 Hz), 1.11 (d, 9H, CH_3,TIPS_, ^3^*J* = 1.4 Hz). UV-visible (CH_2_Cl_2_): λabs (ε) = 422 nm (1,008,610 M^−1^ cm^−1^), 550 nm (45,606 M^−1^ cm^−1^), 589 nm (14,398 M^−1^ cm^−1^). MALDI-TOF MS: *m*/*z* = 2989.9 ([M^+^] calcd. 2990.6).

## 4. Conclusions

Bis-porphyrins with flexible linkers such as uridine or 2′-deoxyuridine pre-organize in a face-to-face conformation and form stable sandwich complexes with a bidentate base such as DABCO. The increased stability can be further stabilized when a dinucleotide linker is used. Such pre-organization increases the association constant by one to two orders of magnitude when compared to the association constant of DABCO with a reference porphyrin. A comparison with rigid tweezers shows a better efficiency of nucleosidic dimers. We thus document the fact that the choice of rigid spacers is not the only way to pre-organize bis-porphyrins, and that some well-chosen nucleosidic linkers offer an interesting option for the synthesis of such devices.

Having the ability to control the special orientation of chromophores in space is a great challenge of modern chemistry. Some of our nucleosidic tweezers—the ones that are easier to synthesize—open great possibilities as supramolecular agents. Despite their recognition capability of classical guests, the chirality and enantio-purity of the nucleosidic linkers pave the way toward the selective complexation of enantio-pure bidentate guests and the resolution of racemates. Work is in progress in this direction.

## References

[1] Lehn J.-M. (1988). Supramolecular Chemistry—Scope and Perspectives Molecules, Supermolecules, and Molecular Devices (Nobel Lecture). Angew. Chem. Int. Ed. Engl..

[2] Lehn J.-M. (2002). Toward self-organization and complex matter. Science.

[3] Fukuzumi S., Saito K., Ohkubo K., Troiani V., Qiu H., Gadde S., D’Souza F., Solladié N. (2011). Multiple photosynthetic reaction centres using zinc porphyrinic oligopeptide-fulleropyrrolidine supramolecular complexes. Phys. Chem. Chem. Phys..

[4] Piet J.J., Taylor P.N., Anderson H.L., Osuka A., Warman J.M. (2000). Excitonic Interactions in the Singlet and Triplet Excited States of Covalently Linked Zinc Porphyrin Dimers. J. Am. Chem. Soc..

[5] Satake A., Kobuke Y. (2005). Dynamic supramolecular porphyrin systems. Tetrahedron.

[6] Choi M., Yamazaki T., Yamazaki I., Aida T. (2004). Bioinspired Molecular Design of Light-Harvesting Multiporphyrin Arrays. Angew. Chem. Int. Ed..

[7] Tomizaki K., Yu L., Wei L., Bocian D.F., Lindsey J.S. (2003). Synthesis of Cyclic Hexameric Porphyrin Arrays. Anchors for Surface Immobilization and Columnar Self-Assembly. J. Org. Chem..

[8] Hwang I.W., Kamada T., Ahn T.K., Ko D.M., Nakamura T., Tsuda A., Osuka A., Kim D. (2004). Porphyrin Boxes Constructed by Homochiral Self-Sorting Assembly: Optical Separation, Exciton Coupling, and Efficient Excitation Energy Migration. J. Am. Chem. Soc..

[9] Mak C.C., Bampos N., Darling S.L., Montalti M., Prodi L., Sanders J.K.M. (2001). A Strategy for the Assembly of Multiple Porphyrin Arrays Based on the Coordination Chemistry of Ru-Centered Porphyrin Pentamers. J. Org. Chem..

[10] Haycock R.A., Yartsev A., Michelsen U., Sundström V., Hunter C.A. (2000). Self-Assembly of Pentameric Porphyrin Light-Harvesting Antennae Complexes. Angew. Chem. Int. Ed..

[11] Bretar J., Gieselbrecht J.-P., Gross M., Solladié N. (2001). Tweezers hosts for intercalation of Lewis base guests: Tuning physico-chemical properties of cofacial porphyrin dimers. Chem. Commun..

[12] Rein R., Gross M., Solladié N. (2004). Adjustable cavity for host-guest recognition in cofacial bis-porphyrinic tweezer. Chem. Commun..

[13] Flamigni L., Talarico A.M., Ventura B., Rein R., Solladié N. (2006). A Versatile Bis-Porphyrin Tweezer Host for the Assembly of Noncovalent Photoactive Architectures: A Photophysical Characterization of the Tweezers and Their Association with Porphyrins and Other Guests. Chem. Eur. J..

[14] Ikeda C., Tanaka Y., Fujihara T., Ishii Y., Ushiyama T., Yamamoto K., Yoshioka N., Inoue H. (2001). Self-Assembly of a Porphyrin Array via the Molecular Recognition Approach: Synthesis and Properties of a Cyclic Zinc(II) Porphyrin Trimer Based on Coordination and Hydrogen Bonding. Inorg. Chem..

[15] Ikeda C., Satake A., Kobuke Y. (2003). Proofs of Macrocyclization of Gable Porphyrins as Mimics of Photosynthetic Light-Harvesting Complexes. Org. Lett..

[16] Kameyama K., Satake A., Kobuke Y. (2004). Light-harvesting composites of directly connected porphyrin–phthalocyanine dyads and their coordination dimers. Tetrahedron Lett..

[17] Schmittel M., Kishore R.S.K. (2004). Tetrakis-heteroleptic Complexation at Porphyrins: A Convenient Route to Diversely Functionalized Aggregates. Org. Lett..

[18] Balaban T.S., Goddard R., Linke-Schaetzel M., Lehn J.-M. (2003). 2-Aminopyrimidine Directed Self-Assembly of Zinc Porphyrins Containing Bulky 3,5-Di-*tert*-butylphenyl Groups. J. Am. Chem. Soc..

[19] Iengo E., Zangrando E., Alessio E. (2003). Discrete Supramolecular Assemblies of Porphyrins Mediated by Coordination Compounds. Eur. J. Inorg. Chem..

[20] Iengo E., Zangrando E., Alessio E., Chambron J.-C., Heitz V., Flamigni L., Sauvage J.-P. (2003). A Functionalized Noncovalent Macrocyclic Multiporphyrin Assembly from a Dizinc(II) Bis-Porphyrin Receptor and a Free-Base Dipyridylporphyrin. Chem. Eur. J..

[21] Sugou K., Sasaki K., Kitajima K., Iwaki T., Kuroda Y. (2002). Light-Harvesting Heptadecameric Porphyrin Assemblies. J. Am. Chem. Soc..

[22] Wojaczynski J., Latos-Grazynski L. (2000). Poly- and oligometalloporphyrins associated through coordination. Coord. Chem. Rev..

[23] Holliday B.J., Mirkin C.A. (2001). Strategies for the Construction of Supramolecular Compounds through Coordination Chemistry. Angew. Chem. Int. Ed..

[24] Robertson A., Shinkai S. (2000). Cooperative binding in selective sensors, catalysts and actuators. Coord. Chem. Rev..

[25] Toma H.E., Araki K. (2000). Supramolecular assemblies of ruthenium complexes and porphyrins. Coord. Chem. Rev..

[26] Leininger S., Oleynuk B., Stang P.J. (2000). Self-Assembly of Discrete Cyclic Nanostructures Mediated by Transition Metals. Chem. Rev..

[27] Anderson H.L., Sanders J.K.M. (1995). Enzyme mimics based on cyclic porphyrin oligomers: Strategy, design and exploratory synthesis. J. Chem. Soc. Perkin Trans..

[28] Vidal-Ferran A., Bampos N., Sanders J.K.M. (1997). Stepwise Approach to Bimetalic Porphyrin Hosts: Spatially Enforced Coordination of a Nickel(II) Porphyrin. Inorg. Chem..

[29] Anderson H.L. (1994). Conjugated Porphyrin Ladders. Inorg. Chem..

[30] Taylor P.N., Anderson H.L. (1999). Cooperative Self-Assembly of Double-Strand Conjugated Porphyrin Ladders. J. Am. Chem. Soc..

[31] Wilson G.S., Anderson H.L. (1999). A conjugated triple strand porphyrin array. Chem. Commun..

[32] Michelsen U., Hunter C.A. (2000). Self-Assembled Porphyrin Polymers. Angew. Chem. Int. Ed..

[33] Gardner M., Guerin A.J., Hunter C.A., Michelsen U., Rotger C. (1999). Self-assembly of zinc aminoporphyrins. New. J. Chem..

[34] Fleischer E.B., Shachter A.M. (1991). Coordination oligomers and a coordination polymer of zinc tetraarylporphyrins. Inorg. Chem..

[35] Slone R.V., Hupp J.H. (1997). Synthesis, Characterization, and Preliminary Host—Guest Binding Studies of Porphyrinic Molecular Squares Featuring *fac*-Tricarbonylrhenium(I) Chloro Corners. Inorg. Chem..

[36] Drain C.M., Nifiatis F., Vasenko A., Batteas J.D. (1998). Porphyrin Tessellation by Design: Metal-Mediated Self-Assembly of Large Arrays and Tapes. Angew. Chem. Int. Ed..

[37] Milic T.N., Chi N., Yablon D.G., Flynn G.W., Batteas J.D., Drain C.M. (2002). Controlled Hierarchical Self-Assembly and Deposition of Nanoscale Photonic Materials. Angew. Chem. Int. Ed..

[38] Reek J.N.H., Schenning A.P.H.J., Bosman A.W., Meijer E.W., Crossley M.J. (1998). Templated assembly of a molecular capsule. Chem. Commun..

[39] Baldini L., Ballester P., Casnati A., Gomila R.M., Hunter C.A., Sansone F., Ungaro R. (2003). Molecular Acrobatics: Self-Assembly of Calixarene-Porphyrin Cages. J. Am. Chem. Soc..

[40] Solladié N., Hamel A., Gross M. (2001). Towards multiporphyrinic alpha-helices with a polypeptidic backbone as system endowed with light harvesting capabilities. Chirality.

[41] Solladié N., Aziat F., Bouatra S., Rein R. (2008). Bis-porphyrin tweezers: Rigid or flexible linkers for better adjustment of the cavity to bidentate bases of various size. J. Porphyr. Phtalocyanines.

[42] Barber J., Andersson B. (1994). Revealing the blueprint of photosynthesis. Nature.

[43] Kühlbrandt W. (1994). Many wheels make light work. Nature.

[44] McDermott G., Prince S.M., Freer A.A., Hawthornthwaite-Lawless A.M., Papiz M.Z., Cogdell R.J., Isaacs N.W. (1995). Crystal structure of an integral membrane light-harvesting complex from photosynthetic bacteria. Nature.

[45] Pullerits T., Sundström V. (1996). Photosynthetic Light-Harvesting Pigment–Protein Complexes: Toward Understanding How and Why. Acc. Chem. Res..

[46] Hirose K. (2001). A Practical Guide for the Determination of Binding Constants. J. Incl. Phenom. Macrocycl. Chem..

[47] Gampp H., Maeder M., Meyer C.J., Zuberbühler A.D. (1986). Calculation of equilibrium constants from multiwavelength spectroscopic data—IV: Model-free least-squares refinement by use of evolving factor analysis. Talanta.

[48] Gampp H., Maeder M., Meyer C.J., Zuberbühler A.D. (1985). Calculation of equilibrium constants from multiwavelength spectroscopic data—I: Mathematical considerations. Talanta.

[49] Mak C.C., Bampos N., Sanders J.K.M. (1998). Metalloporphyrin Dendrimers with Folding Arms. Angew. Chem. Int. Ed..

[50] Ballester P., Costa A., Castilla A.M., Deyà P.M., Frontera A., Gomila R.M., Hunter C.A. (2005). DABCO-Directed Self-Assembly of Bisporphyrins (DABCO=1,4-Diazabicyclo[2.2.2]octane). Chem. Eur. J..

[51] Hunter C.A., Meah M.N., Sanders J.K.M. (1990). DABCO-metalloporphyrin binding: Ternary complexes, host-guest chemistry and the measurement of π–π interactions. J. Am. Chem. Soc..

[52] Anderson H.L., Hunter C.A., Meah M.N., Sanders J.K.M. (1990). Thermodynamics of induced-fit binding inside polymacrocyclic porphyrin hosts. J. Am. Chem. Soc..

[53] Kim H.J., Bampos N., Sanders J.K.M. (1999). Assembly of Dynamic Heterometallic Oligoporphyrins Using Cooperative Zinc–Nitrogen, Ruthenium–Nitrogen, and Tin–Oxygen Coordination. J. Am. Chem. Soc..

[54] Hunter C.A., Tregonning R. (2002). Modular assembly of porphyrin sandwiches as potential hosts. Tetrahedron.

[55] Mak C.C., Pomeranc D., Montelti M., Prodi L., Sanders J.K.M. (1999). A versatile synthetic strategy for construction of large oligomers: Binding and photophysical properties of a nine-porphyrin array. Chem. Commun..

